# PREBASELINE SELECTION BIAS IN THE ESTIMATED EFFECT OF T2D ON COGNITIVE DECLINE: CROSS-NATIONAL SIMULATIONS

**DOI:** 10.1093/geroni/igae098.2081

**Published:** 2024-12-31

**Authors:** Pengyuan (Kelvin) Zhang, Sneha Mani, Lindsay Kobayashi, Alden Gross, Jennifer Weuve, Ryan Andrews

**Affiliations:** University of Michigan, Okemos, Michigan, United States; Johns Hopkins Bloomberg School of Public Health, Baltimore, Maryland, United States; University of Michigan, Ann Arbor, Michigan, United States; Johns Hopkins Bloomberg School of Public Health, Baltimore, Maryland, United States; Boston University, Boston, Massachusetts, United States; Boston University, Boston, Massachusetts, United States

## Abstract

Selection bias is a common threat to the validity of effect estimates, including those of various exposures on late-life cognitive outcomes. Although selection bias can arise for many reasons, a challenging subtype is driven by differential mortality in a study’s source population prior to baseline. In other words, individuals who survive long enough to be eligible for studies of late-life cognition are systematically different from those who do not. Because data are not collected on individuals who do not survive to study enrollment, analytic methods to address selection bias cannot be applied. Furthermore, this “pre-baseline survival” bias makes cross-national comparisons difficult, as different countries have differing mortality rates and determinants of mortality. Using the example of cross-national comparisons of the effect of type-2 diabetes on cognitive decline, we demonstrate how simulations can help quantify pre-baseline survival bias. We simulated large (N=300,000) country-specific cohorts of adults in the US, India, and China under seven causal scenarios governing the relationships between type-2 diabetes, pre-baseline survival, and cognitive decline. After assigning each simulated participant a vital status (dead/alive by a certain age), we recruited 2,000 country-specific study samples of 2,000 “survivors” and estimated the association between type-2 diabetes and cognitive decline within these cohorts. We found that even under relatively mild differences in pre-baseline survival, the cross-national effect of diabetes on cognitive decline could be underestimated by up to 34%. To assist others with conducting similar simulation studies, we developed a user-friendly platform based on the R-Shiny package.

